# Analysis of Influencing Factors in the Preparation of Mullite Whiskers from Recovering Silicon-Rich Waste under Low-Temperature Conditions

**DOI:** 10.3390/nano13071143

**Published:** 2023-03-23

**Authors:** Xiaohua Gu, Peiquan Chen, Tong Wang, Siwen Liu, Shangwen Zhu, Yanwei Zhu, Yan Liu

**Affiliations:** 1 School of Energy and Building Environment, Guilin University of Aerospace Technology, Guilin 541004, China; 2 State Key Laboratory for Modification of Chemical Fibers and Polymer Materials, College of Materials Science and Engineering, Donghua University, Shanghai 200051, China; 3 School of Material Science and Engineering, Qiqihar University, Qiqihar 161006, China; 4 College of Innovative Material and Energy, Hubei University, Wuhan 430062, China

**Keywords:** silicon-rich, mullite whiskers, low temperature, sintering temperature, keep-warm time

## Abstract

A large amount of catalyst waste containing silicon is deposited or buried every year, resulting in serious environmental pollution and a waste of resources. In this paper, a method to prepare mullite whiskers by recycling silica-rich waste under low-temperature conditions was investigated. The effects of raw materials, sintering temperature, catalyst addition, holding time and co-solvent addition on the structure, morphology and phase transition of the synthesized whiskers were investigated and characterized with SEM, XRD, TEM, TG and DTA. The results show that the addition of 10% Na_2_SO_4_ as the liquid-phase mass transfer medium could effectively improve the crystallization efficiency of mullite whiskers, while providing an ideal living environment for the growth of whiskers. The crystallinity and uniformity of mullite were positively correlated with the addition of aluminum fluoride trihydrate and the holding time, respectively. The growth law and conditions of mullite whiskers are discussed, and the optimal growth process conditions of mullite whiskers were optimized. The optimal conditions for mullite whiskers were determined as follows: the addition of aluminum fluoride is 5 wt%, the sintering temperature is 825 °C, and the holding time is 5 h at the time of sintering. This work offers new prospects for the industrial production of mullite whiskers from recycled silica-rich waste.

## 1. Introduction

The continuous development of science and technology and the increasing energy consumption in recent years have resulted in the production of many solid wastes in the chemical and petroleum industries, which are usually sent to landfills. Silica-rich wastes are industrial wastes from refinery catalyst plants and are usually mixed with many of the beneficial components of the natural oxides of catalysts, which makes them chemically quite active. Most of the components of silica-rich wastes are soluble in water or slightly soluble in water, causing white water pollution. Because of its rich mineral composition, silica-rich waste has a high potential for industrial exploitation. To successfully develop methods to recover silica-rich wastes, it is essential to understand their composition and characteristics. The molecular formula of silica-rich waste is mSiO_2_-nAl_2_O_3_-xH_2_O, which is chemically similar to kaolinite and sapphire minerals and can be a potential source of material for the preparation of mullite whiskers.

Among the inorganic whiskers, mullite whiskers belong to the composition series of orthogonal aluminosilicates with a general composition of Al_2_(Al_2+2x_Si_2−2x_)O_10−x_, x between about 0.2 and 0.9 (about 55 to 90 mol% Al_2_O_3_) [1]. They effectively improve the mechanical properties of materials and prevent the development of cracks by significantly increasing flexural strength and fracture toughness, which enables them to be widely used in the field of ceramic and metal matrix composites [2,3,4,5,6]. The crystallographic direction parallel to the c-axis, in which mullite crystals grow much faster than in other directions, is advantageous for the synthesis of mullite whiskers with a high degree of orientation [7,8]. Moreover, the fact that mullite is formed by dissolution-precipitation, which is rare under natural conditions, leads to the common synthesis of mullite whiskers by artificial means [9,10]. Based on the above principles, most researchers have prepared mullite whiskers by adding an excess of single transition metal oxides as catalysts, such as WO_3_ [11], MoO_3_ [12], V_2_O_5_ [13], P_2_O_5_ [14], B_2_O_3_ [15], etc. Their effect is to cause the rapid formation of a glassy phase of SiO_2_, leading to the ability of alumina to dissolve rapidly in this glassy phase and finally in the form of mullite whiskers [16,17,18]. All of these methods lower the crystallization temperature of mullite and promote the growth rate of mullite in the direction of crystallization along the c-axis. Hua et al. [19] successfully obtained mullite whiskers by sintering at 1350 °C for 1 h using construction waste and Al_2_O_3_ powder as raw materials by adding a total of 12 wt% AlF_3_ and 4 wt% MoO_3_. Xu et al. [20] used an in situ bonding technique with silicon carbide particles as the skeleton material, fine kaolin and α-Al_2_O_3_ powder as the binder, activated carbon as the pore-forming agent and MoO_3_ as the additive at 1350 °C. The mullite-whisker-reinforced porous silicon carbide film scaffolds were prepared by pressureless sintering at 1520 °C. It can be seen that most of the whiskers were sintered at high temperatures, between 1200–1400 °C, and the raw materials used need to be purchased, which is not economical. Although Li et al. [21] synthesized mullite whiskers in situ using recycled cinder as raw material and MoO_3_ and AlF_3_ as additives, the sintering temperature reached 1300 °C, which is still costly in comparison. However, the synthesis temperature of these methods is >1200 °C, which is not conducive to industrial production. Researchers have turned to industrial waste for the low-cost, low-temperature preparation of mullite whiskers. Still, the mass production of high-quality mullite whiskers from industrial waste is a major challenge in today’s world. Nowadays, it is still the trend to develop mullite industrialization by recycling industrial waste, such as recycling industrial aluminum slag [22,23], fly ash [24,25], industrial gangue [26,27], and photovoltaic silicon waste [28,29] to produce mullite whiskers. Deng et al. [9] prepared mullite-whisker-reinforced lightweight porous materials using fly ash and calcined bauxite as raw materials and AlF_3_·3H_2_O as an additive using the particle stabilization method. Li et al. [30] prepared self-reinforced porous mullite ceramics using fly ash, different aluminum sources (Al(OH)_3_ and Al_2_O_3_) and additive AlF_3_ as raw materials using the starch consolidation method. It has been observed that the addition of rare earth oxides to aluminosilicates results in the formation of a glassy phase that can promote nucleation and whisker growth [31]. Components of La_2_O_3_ or CeO_3_ in Si-rich scrap can be used as part of the catalyst, helping to develop better whiskers and reduce the cost of artificially adding rare earth oxides. It is important to develop a method to produce mullite whiskers in high volume at low cost to expand their applications. As far as we know, however, there are few reports on recycling silica-rich waste in the preparation of mass-produced mullite whiskers. Therefore, it is urgent to solve the problem of converting silica-rich scrap into a toughened material for a wide range of applications.

For this paper, mullite whiskers were synthesized by a simple and inexpensive method using silica-rich waste as the main raw material, and the effects of rare earth oxides in raw material, sintering temperature, aluminum fluoride addition, holding time and sodium sulfate addition on the whiskers were analyzed to explore the growth pattern and conditions of mullite whiskers. This is of great significance for the industrial mass production of mullite whiskers.

## 2. Experimental Raw Materials and Preparation Methods

### 2.1. Experimental Materials

In this study, silicon-rich waste (purchased from Zhongxiao New Material Technology Co., Ltd., Zibo, Shandong, China) and Al_2_(SO_4_)_3_·18H_2_O (Tianjin Damao Chemical Reagent Co., Ltd., Tianjin, China, analytical purity, 99.0%) were used as the main raw materials for the synthesis of mullite whiskers. As the source of aluminum for this experiment, the aluminum ions produced by the high-temperature decomposition of aluminum sulfate have high activity. AlF_3_·3H_2_O (Sinopharm Chemical Reagent Co., Ltd., Shanghai, China, chemical purity, 98.0%) was selected as the crystallization catalyst, Na_2_SO_4_ (Tianjin Tianli Chemical Reagent Company, Tianjin, China, analytically pure, 99.0%) was selected as the flux. On the basis that the waste itself contains sodium sulfate, an appropriate amount of Na_2_SO_4_ molten salt was added to generate a larger liquid crystal growth environment. AlF_3_·3H_2_O and Na_2_SO_4_ were introduced to construct a vapor–liquid–solid reaction mechanism, which would assist the development and anisotropic growth of mullite seeds and constructing gas-phase mass transfer and liquid-phase mass transfer processes. Compared with the single liquid-phase reaction and gas-phase reaction, the two mass transfer processes can promote the nucleation of mullite and solve the problem of a low yield of mullite whiskers. The chemical composition of industrial waste silicone aluminum glue is shown in Table 1 and Table 2 shows the chemical composition (wt%) analysis of each component of the pretreated silica-rich waste powder after removal of rare earth oxides. The tail gas and wastewater involved in this experiment had been purified by a special treatment facility.

### 2.2. Synthesis Steps of Mullite Whiskers

**Figure 1:**
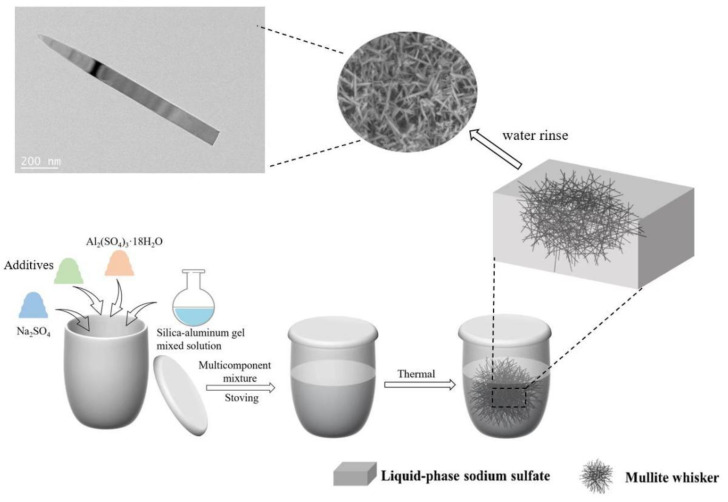
Shows the flow chart for the preparation of mullite whiskers.

Firstly, a mixed suspension of industrial silica-rich waste, Al_2_(SO_4_)_3_·18H_2_O, aluminum fluoride trihydrate and anhydrous sodium sulfate powder in Al:Si:Na molar ratio of 3:1:0.2 was placed in a ball mill jar, mixed in a ratio of ball:material:water of 4:2:1, and run wet with a ball mill (Ball Mill SBM-7, Xingyang City, Henan Province, Micro Powder Equipment Manufacturing Plant) with the speed of 300 rpm for 6 h, and then the suspension was poured into a corundum crucible and dried in a 110 °C oven for 12 h. The corundum crucible was then covered with a lid and fired in an electric furnace at 700–1000 °C at a rate of 5 °C/min and held in this temperature range for 5 h. The samples were cooled naturally to room temperature in the oven and rinsed 5–7 times with deionized water. Figure 1 shows the flow chart for the preparation of mullite whiskers. All HF, SO_2_ and SO_3_ produced during the reaction were collected by a special exhaust gas treatment unit at the exhaust port of the furnace.

### 2.3. Characterization Methods

Industrial silicon-rich waste was analyzed for its chemical composition using a rapid silicate chemical composition tester (GKF-IV, Xiangtan Xiang Yi Instrument Co., Ltd., China). An X-ray diffractometer (DX-2700, Dandong Haoyuan Instrument Co., Dandong, China) was used to analyze the phase composition of samples calcined at various temperatures with Cu Kα radiation λ = 0.154 nm, which is produced by 30 mA and 40 kV, and scanned at a rate of 10°/min in the range of 10–90° with a step size of 0.03°. Specimen bonds at different temperature reaction stages were identified by Fourier transform infrared spectroscopy (FT-IR, Spectrum One, Perkin Elmer, Waltham, MA, USA) with wave numbers in the range of 4000–400 cm^−1^. Specimens were heated from room temperature to 1000 °C at 5 °C/min in air. Precipitate thermal decomposition processes were determined via differential thermal analysis and thermogravimetric analysis (DTA-TG, STA 449 F3 Jupiter, NETZSCH AG, Bavaria, Germany). Mullite whisker microstructures were observed by scanning electron microscopy (SEM, Quanta 250, Thermo Fisher Co., 81 Wyman Street, Waltham, MA, USA) under an accelerating voltage of 10 kV. Transmission electron microscopy (TEM, Tecnai G2 F20) was used to observe the microscopic morphology of mullite whiskers, and selected area electron diffraction (SAED) was used to analyze the crystallographic structure of the samples.

## 3. Results and Discussion

In this paper, we mainly compare the differences between the whiskers made by using raw materials containing rare earth elements and those without, and the beneficial effects of using Na_2_SO_4_ as a flux, and investigate the effects of the addition of aluminum fluoride trihydrate, sintering temperature and holding time on the crystallinity and homogeneity of mullite whiskers.

### 3.1. Rare Earth Oxides in Raw Materials

**Figure 2:**
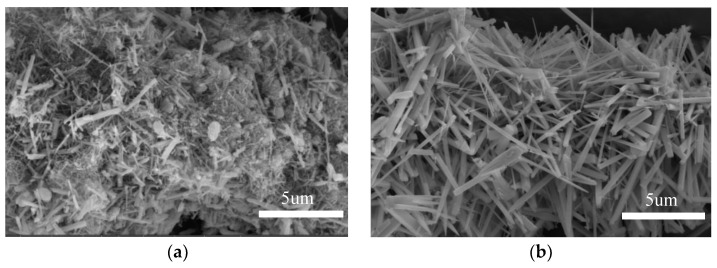
SEM images of mullite whiskers prepared from feedstock without rare earth oxides (**a**) and from feedstock with rare earth elements (**b**).

**Figure 3:**
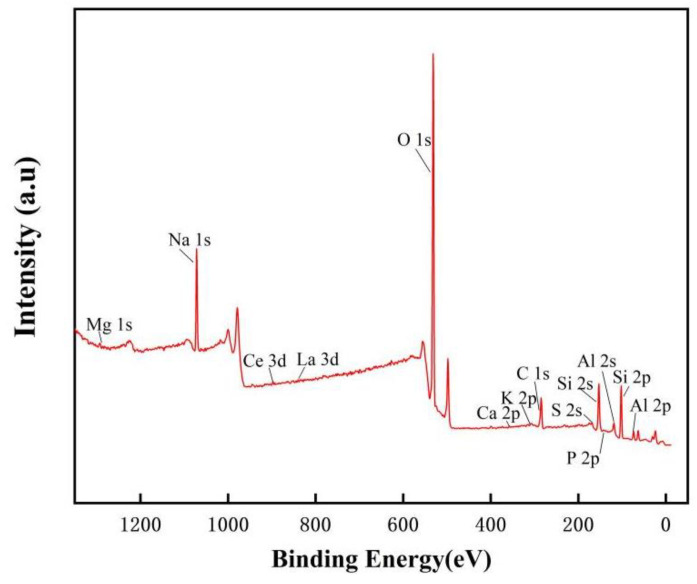
XPS of mullite whiskers after washing and drying.

Efforts have been made to compare whisker phases prepared from feedstocks with and without rare earth oxides during whisker preparation, and it was found that the presence of rare earth oxides has an essential effect on the growth of whiskers [31]. Figure 2a illustrates SEM images of the whiskers prepared using raw materials without rare earth oxides, and Figure 2b shows SEM images of the whiskers containing rare earth elements. It is evident that the mustache prepared using raw materials without rare earth oxides is more disordered than the whiskers containing rare earth elements. It can be noted that rare earth ions exhibit crystalline nucleation activity with increasing atomic number of rare earth elements, as the ionic radius decreases and the ionic field strength increases. The ionic radii of La^3+^, Ce^3+^, and Ce^4+^ are 103.2 pm, 102 pm, and 87 pm, respectively, which are much larger than those of Al^3+^ (53.5 pm) and Si^4+^ (40 pm), and it is likely that these rare earth elements will not enter the crystal due to the size difference [31]. Thus, they are removed during the drying and washing process after the whisker preparation is completed, as can be seen from the XPS images of the prepared mullite whiskers (as shown in Figure 3), where La and Ce are removed.

Rare earth elements show good dispersion properties in improving catalyst performance and the electrodeposition process [32]. Industrial silica-rich waste containing La and Ce elements is used as the raw material for whisker preparation, which can avoid agglomeration during crystal growth. Further, La and Ce will significantly refine the grains and increase the number of grain boundaries, so that the mullite whiskers are well aligned and grow uniformly with better dispersion, which can be verified in both SEM and TEM images. 

At the sintering temperature, the rare earth elements in the raw material are very active. On the one hand this will reduce the surface macroscopic stress of the product, on the other hand it will reduce the diffusion resistance between atoms during the sintering process, thus speeding up the sintering process and lowering the sintering temperature [33], which reduces the mullite preparation temperature from the conventional 1100–1500 °C to 720 °C, which is reflected in the XRD spectrum of the resulting whiskers [34]. However, due to the large atomic weight of La and Ce, the movement process is more difficult compared to elements such as Al and O, thus hindering the crystallization process and increasing the crystallization time, enabling the crystals to have enough time to grow intact and avoid lattice defects [35,36,37]. Additionally, the effect of metamorphic inclusions and reactive oxygen on the surface of the reduced particles possessed by rare earth elements will improve the strength and especially the toughness of the resulting whiskers.

### 3.2. V–L–S Growth Atmosphere Influence

In this paper, mullite whiskers were prepared by using silicon-rich waste as the main raw material, supplemented with aluminum sulfate powder as the supplementary aluminum source, aluminum fluoride as the whisker catalyst, and low melting point sodium sulfate as the sintering flux. Since the high-temperature molten salt is in liquid phase, the reaction system exists in three states: solid, gas and liquid, and the mullite whisker generation follows the V–L–S growth mechanism. In the first stage, the gas-phase material diffuses into the catalyst droplet; in the second stage, the gas-phase material reacts with the droplet on the droplet to generate a new phase; in the third stage, the new phase generated by the reaction is transported through the droplet to the interface, and at the same time, if there are gas-phase by-products, the by-products will diffuse back to the gas phase; in the fourth stage, the new phase is deposited at the solid–liquid interface and finally grows into a solid whisker. Each stage of growth atmosphere atomic transport and diffusion will seriously affect the overall whisker growth rate [8]. The chemical reaction of mullite whiskers can be expressed by the following equations [3,8]:

2AlF_3_ (g) + O_2_ (g) → 2AlOF (g) + 4F (g) (1)

Al_2_O_3_ (s) + 2F (g) → 2AlOF (g) + 0.5O_2_ (g) (2)

SiO_2_ (s) + 4F (g) → SiF_4_ (g) + O_2_ (g) (3)

6AlOF (g) + 2SiF_4_ (g) + 3.5O_2_ → 3Al_2_O_3_·2SiO_2_ (s) + 14F (g) (4)

**Figure 4:**
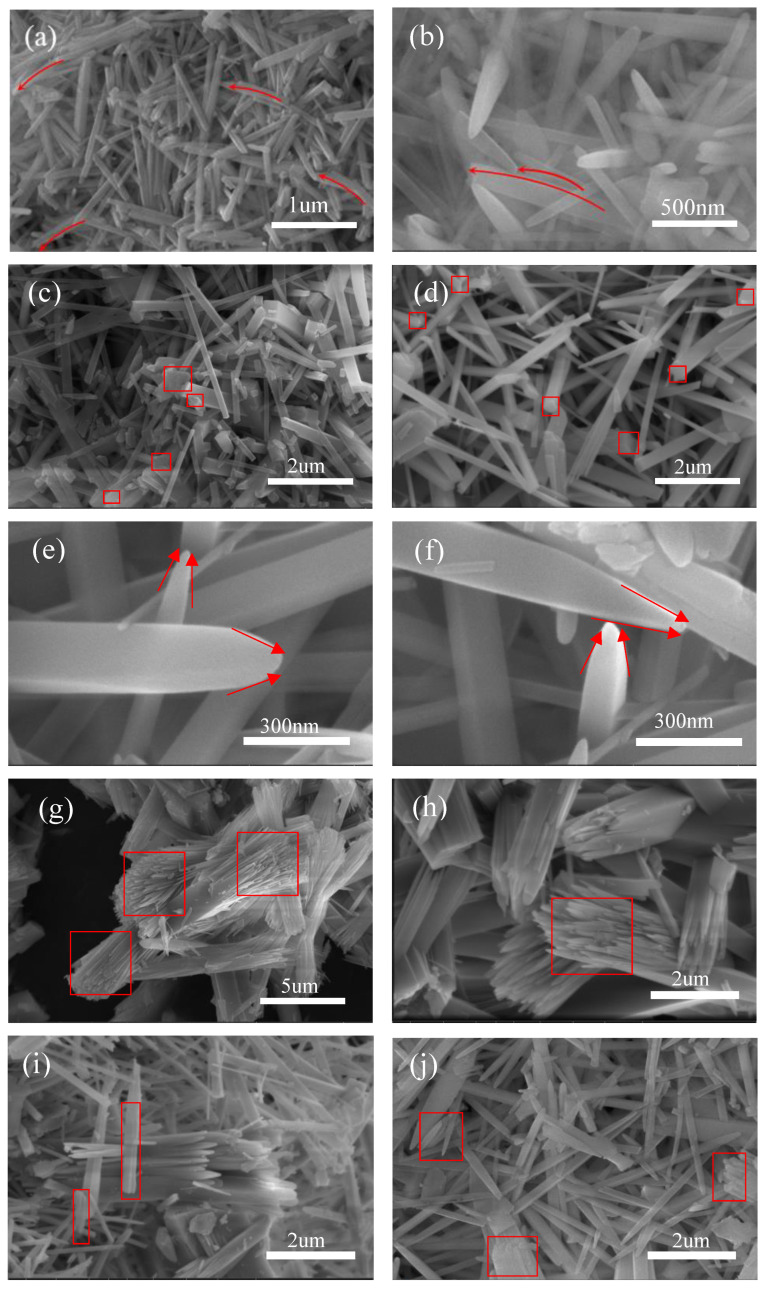
SEM images of various special morphological structures of mullite whiskers: (**a**,**b**) Whisker twist; (**c**,**d**) whisker tip droplets; (**e**,**f**) the top angles of the whiskers; (**g**,**h**) secondary growth of whiskers; (**i**,**j**) stacking fault.

The structure of various special morphologies of the prepared mullite whiskers is shown in Figure 4. The deformation phenomenon occurring in the whiskers, as shown in Figure 4a,b, is due to the lattice deformation caused by helical dislocations, resulting in the radial deformation of the mullite whiskers. The melts formed by residual droplets on top of the mullite whiskers are shown in Figure 4c,d, which are typical of the whiskers after V–L–S growth, and the wedge-shaped structure is due to the different deposition rates of the gas-phase components during the growth process, resulting in a change in the composition of the catalytic droplets. As shown in Figure 4e,f, the sharp and smooth whisker tips, clear and bright angles, and uniform whisker surfaces indicate that the whiskers follow the V–S growth mechanism. This is typically characterized by a gas–solid reaction, in which a gas phase generated by the reaction components at high temperatures passes to the surface of the nucleus when the crystal is nucleated, leading to its directed growth into whiskers. The secondary growth of the whiskers can be observed in Figure 4g,h, which originates from the spiral dislocations that occur during the axial growth of the whiskers, i.e., the diffusion of atoms from the top of the whiskers to the top growth, causing the mullite whiskers to grow along the axial direction. The whisker growth stops when the dislocation is far from the whisker tip due to the insufficient supply of atoms at the tip. Figure 4i,j show the growth step (stacking layer dislocation) left by the whisker growth process. This development step is different from the helical pattern left by axial helical dislocation growth which is located on one side of the whisker, and its growth mechanism is attributed to parallel extensional dislocations on the tilted grain boundaries.

Based on these phenomena, it can be inferred that the stacking layer dislocations on the mullite whisker surface and the helical convex grooves at the whisker tip in a special growth environment suggest the appearance of tough dislocations and helical dislocations in the lattice during the mullite whisker growth stage. Therefore, the generation of perfect whiskers requires a continuous and stable supply of atoms to grow uniformly sized and complete whiskers, which requires perfect control of the atomic diffusion rate during the whisker preparation process to be uniform and stable, and all three phases of vapor–liquid–solid to ensure a continuous and stable atomic migration process in order to ensure the same deposition rate of gas phase components and the stable deposition of new phases at the solid–liquid interface to form mullite whiskers with perfect surface morphology. Therefore, we will discuss later how the whisker catalyst aluminum fluoride and the liquid phase sodium sulfate, which provides the high temperature molten salt, affect and control the growth atmosphere on the atomic diffusion rate, thus dissecting the important influence of the growth environment on the nucleation and growth of mullite whiskers.

### 3.3. Effect of Flux Na_2_SO_4_

Na_2_SO_4_ is currently the most commonly used flux in the preparation of mullite whiskers, and its addition can effectively reduce the sintering temperature of the reaction [38]. The addition of Na_2_SO_4_ during the preparation process, which reacts with aluminum compounds to form Al_2_(SO_4_)_3_, results in the following chemical reactions [38,39]:

(5) $${\mathrm{Al}}_{2}({\mathrm{SO}}_{4}{)}_{3}\cdot 18H_{2}O\overset{120\;{}^{\circ}C}{\to }{\mathrm{Al}}_{2}({\mathrm{SO}}_{4}{)}_{3}\cdot 3H_{2}O\;(s)+15H_{2}O\;(g)$$

(6) $${\mathrm{Al}}_{2}({\mathrm{SO}}_{4}{)}_{3}\cdot 3H_{2}O\overset{330\;{}^{\circ}C}{\to }{\mathrm{Al}}_{2}({\mathrm{SO}}_{4}{)}_{3}\;(s)+3H_{2}O\;(g)$$

(7) $${\mathrm{Al}}_{2}({\mathrm{SO}}_{4}{)}_{3}\overset{642\;{}^{\circ}C}{\to }\gamma -{\mathrm{Al}}_{2}O_{3}\;(s)+2{\mathrm{SO}}_{3}\;(g)$$

This is because the electrolyte forms free cations and anions when it burns above its melting point. According to this principle, free Al^3+^ is first formed in the liquid before the decomposition of Al_2_(SO_4_)_3_, which is converted to amorphous Al_2_O_3_ above 700 °C. As Al_2_(SO_4_)_3_ decomposes, γ − Al_2_O_3_ and silicon-rich waste gradually form mullite crystals by the V–L–S reaction mechanism in the liquid-phase reaction system provided by the fluorine atmosphere of aluminum fluoride and sodium sulfate. Meanwhile, the liquid phase matrix growth atmosphere of sodium sulfate accelerates the diffusion rate of the medium and atoms, and the low viscosity of the liquid phase accelerates the reaction rate at the liquid/solid phase boundary, which in turn has a significant fluxing effect on the Al_2_O_3_-SiO_2_ system, thus melting at a lower temperature and promoting the growth of mullite crystals and the nucleation growth of mullite crystals [39].

**Figure 5:**
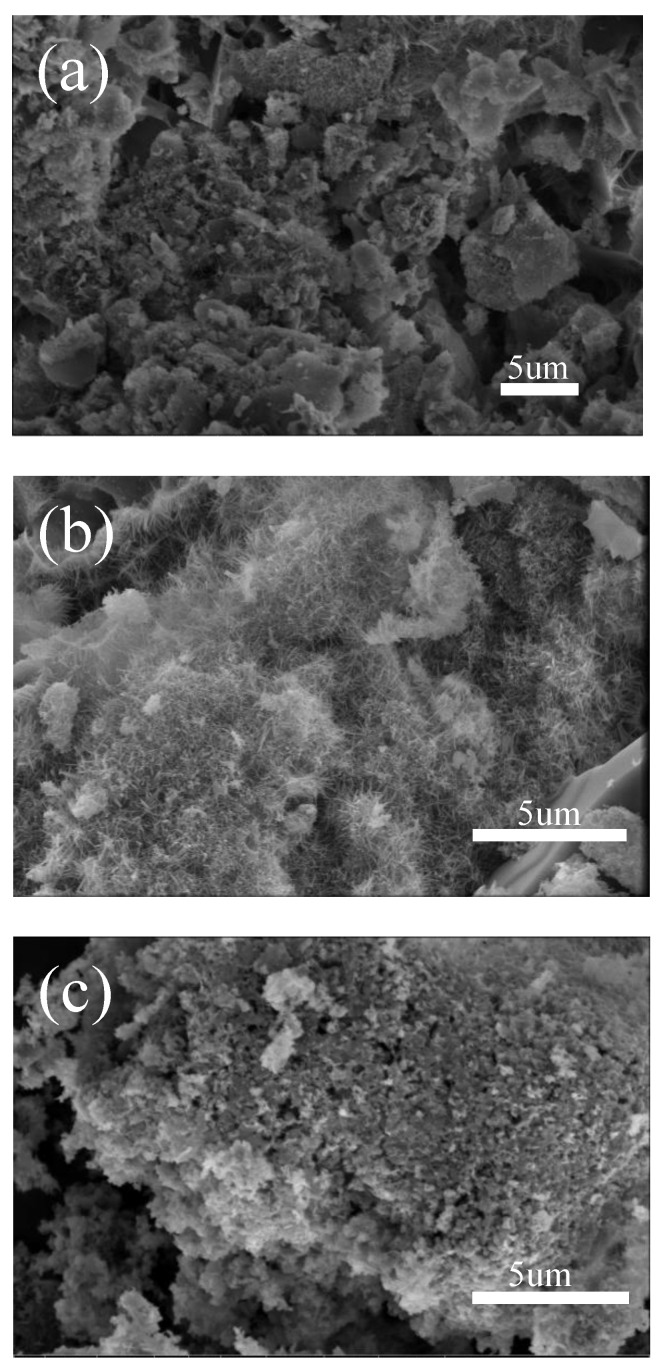
SEM images of whiskers prepared by adding (**a**) 5%, (**b**) 10%, (**c**) 15% of Na_2_SO_4_.

In addition to Na_2_SO_4_ as a mass transfer medium, it also provides a homogeneous external environment for the growth of whiskers, and there is a certain number of helical dislocations in the axial direction of mullite daisy crystals, which provide conditions for the growth of whiskers as a growth source so that the whiskers can grow smoothly. Figure 5a shows an SEM image of mullite whiskers prepared by adding 5% Na_2_SO_4_, the content of which is obviously lower than that of whiskers prepared by adding 10% Na_2_SO_4_ in Figure 5b, which is because the sintering process is affected by too little flux content, which is not conducive to the nucleation and growth of whiskers. Figure 5c shows an SEM image of mullite whiskers prepared by adding 15% Na_2_SO_4_. While higher flux additions are beneficial to whisker growth, excessive flux levels can lead to formation in the form of silicates, which severely affects whisker production. In comparison, the highest whisker content is shown in Figure 5b, and accordingly the Na_2_SO_4_ addition was determined to be 10% for the preparation of mullite whiskers in this paper.

### 3.4. Sintering Temperature

A suitable sintering temperature will provide good thermal dynamics for the growth of nuclei. If the sintering temperature is low, the grain development is incomplete and does not produce high-quality whiskers, which will also reduce the yield of whiskers. Too high a sintering temperature will make grain growth too fast, which will lead to the decrease of the whisker aspect ratio or lead to the final product containing corundum, which will also lower mullite whisker purity. We used different sintering temperatures of 700, 720, 750, 800, 825, 850, 875, and 900, respectively. In the following, the effects of sintering temperature on mullite whiskers analyzed from XRD plots, FTIR absorption spectra, and TG-DTA curves are described; the microstructures of whiskers grown at the optimum temperature were also observed by SEM and TEM.

**Figure 6:**
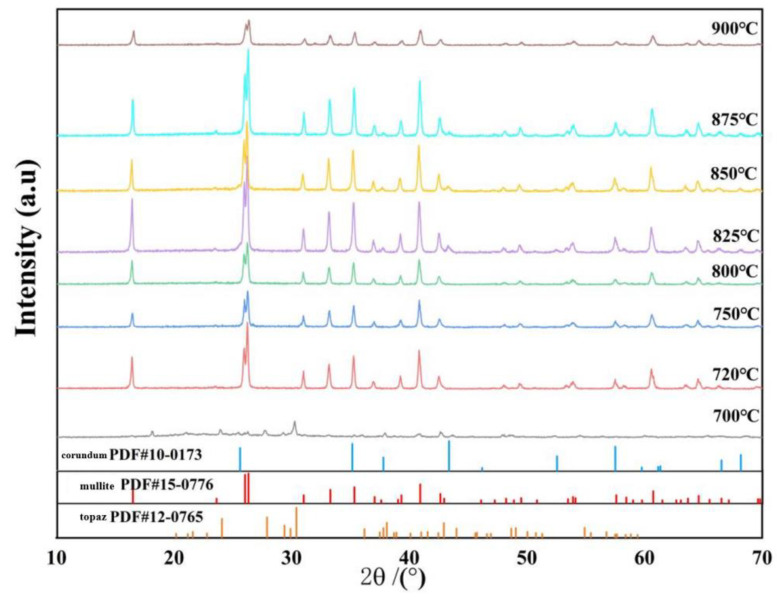
XRD patterns of samples sintered at 700–900 °C for 5 h.

XRD patterns of the samples sintered at 700 to 900 °C for 5 h are shown in Figure 6. It can be seen from the XRD curves that as the temperature increases, the sample undergoes a series of phase transformation processes leading to the lithification temperature of mullite initially occurring at 720 °C. The main crystal diffraction peaks of the samples fired between 720–900 °C are all mullite (Al_6_Si_2_O_13_, plagioclase, (PDF#15-0076), and no impurity phase generation is ever observed throughout the phase evolution process, indicating that the studied compositions are all converted to mullite stoichiometry. On the contrary, at a lower temperature (700 °C), although no crystal diffraction peaks of mullite were detected, diffraction peaks of topaz phase were presented (Al_2_[SiO_4_](F, OH)_2_, orthorhombic, (PDF#12-0765)). However, the diffraction peak intensity of the mullite phase of the sintered sample at 750 °C is lower than that at 720 °C, which may be due to the secondary growth of columnar mullite from the top at 750 °C, and the secondary growth occurs in the interior, forming many fine mullite whiskers with poor crystallinity, resulting in the decrease of the diffraction peak intensity, which is similar to results described in previous work [38,39]. The intensity of the diffraction peak of the mullite phase increases rapidly when the sintering temperature of the sample is increased from 800 to 875 °C. In contrast, a faint diffraction peak of corundum (Al_2_O_3_, hexagonal, (PDF#10-0173)) appears in the sample sintered at 900 °C.

**Figure 7:**
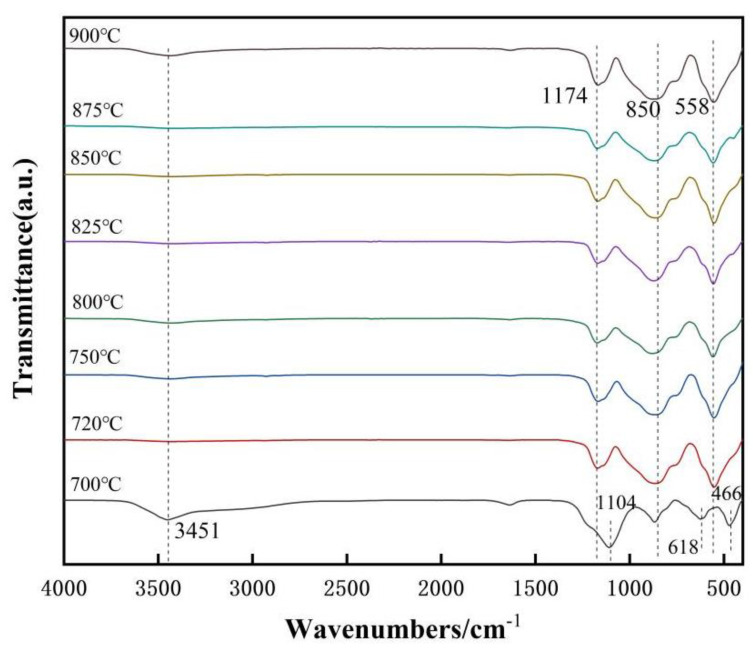
FTIR spectra of samples sintered at 700–900 °C for 5 h.

The FTIR analysis was performed to verify the group transformation process of mullite at the various reaction temperatures, as shown in Figure 7. For the sintering sample at 700 °C, the most pronounced peaks are located at 1104 cm^−1^, 618 cm^−1^ and 466 cm^−1^. These peaks are usually attributed to the stretching vibrations of the Si-O-Si, AlO_6_ and Si-O-Si bonds. By examining the IR spectra from 720–900 °C, it was found that they show spectral bands with typical mullite characteristics near 558 cm^−1^, 850 cm^−1^, and 1174 cm^−1^, corresponding to the vibrations of AlO_6_, AlO_4_ and SiO_4_. This suggests that mullite is produced from 720 °C and undergoes a reciprocal transition from amorphous to crystalline structure, which leads to the recombination of atoms and the accompanying orderly distribution of Al^3+^ and Si^4+^ into the structure, eventually forming mullite whiskers [40,41].

**Figure 8:**
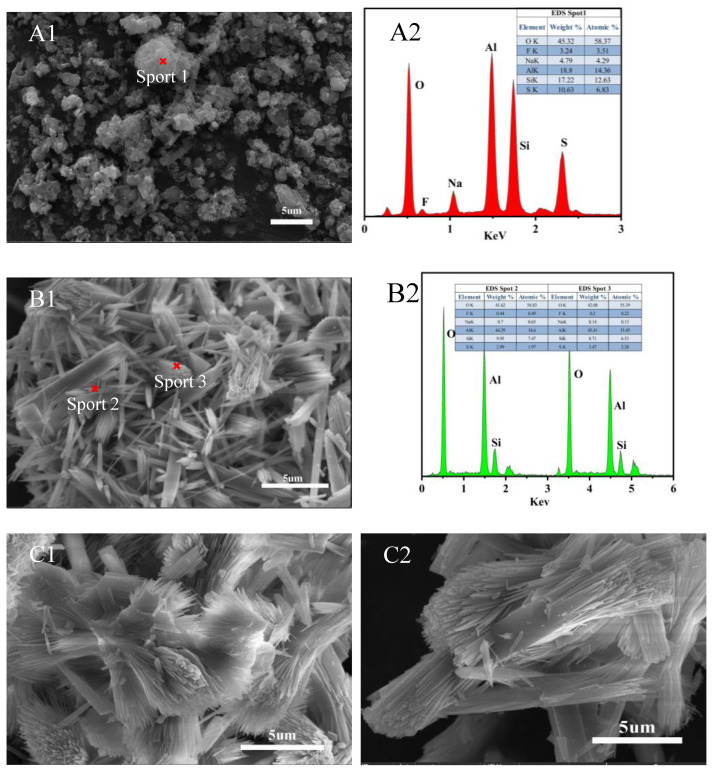
SEM images of mullite whiskers sintered at 700–750 °C. (**A1**) 700 °C; (**A2**) the EDS elemental analysis pattern of the corresponding point of (**A1**); (**B1**) 720 °C; (**B2**) the EDS elemental analysis pattern of the corresponding point of (**B1**); (**C1**,**C2**) 750 °C.

**Figure 9:**
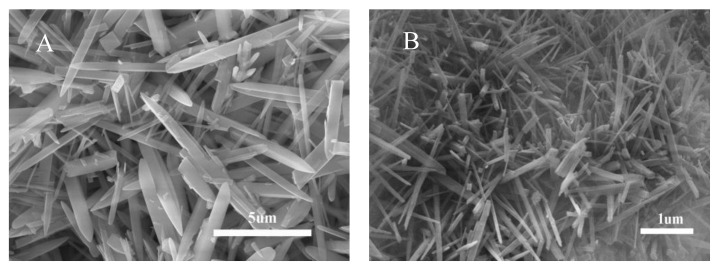

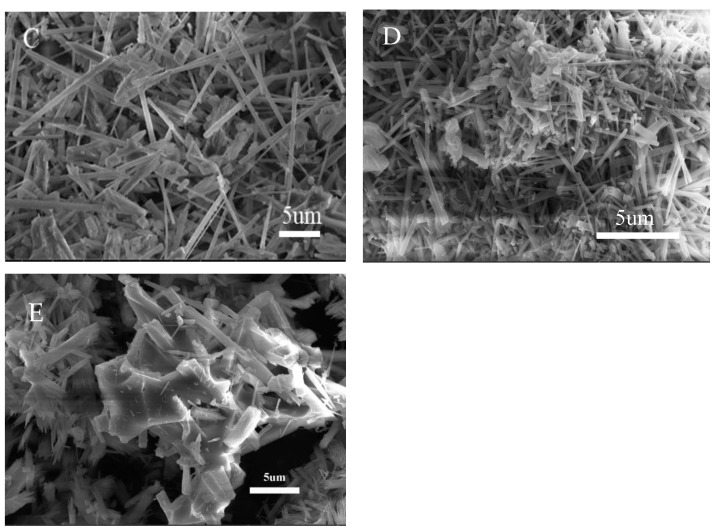
SEM images of mullite whiskers sintered at 800–900 °C. (**A**) 800 °C; (**B**) 825 °C; (**C**) 850 °C; (**D**) 875 °C; (**E**) 900 °C.

The morphology of mullite whiskers obtained by sintering AlF_3_·3H_2_O assisted Na_2_SO_4_ molten salt at 700 to 900 °C is shown in Figure 8. Mullite whiskers could not be detected in the sample sintered at 700 °C as shown in Figure 8A1, but the glass phase is considered to be produced by solidification of the liquid phase rich in SiO_2_ and Na_2_SO_4_ based on the elemental composition of Al, Si, O, S, F and Na as shown by EDS in Figure 8A2, while the fine particles in the glass phase are undissolved Al_2_O_3_, which matches previous research [42,43]. As the reaction temperature increases to 720 °C (Figure 8B1), i.e., the energy in the system just reaches the lowest critical nucleation barrier for mullite formation, the pre-formed mullite grains keep accumulating and growing into columnar mullite whiskers, and the whiskers begin to form. The secondary growth pattern of mullite whisker formation into columnar tops is shown in Figure 8B1. Based on the elemental composition analysis of Al, Si and O in the EDS of Figure 8B2, it is demonstrated that the columnar and needle-like crystals in the figure are basically consistent with the stoichiometry of mullite. Mullite whiskers appear as a fan-shaped structure as shown in Figure 8C1, where both the whisker surface and the adjacent whiskers originate from the decomposition process of the original whiskers. The columnar mullite is decomposed into multiple uniformly fine mullite whiskers from the top to the middle of the whiskers until the mullite whiskers are peeled off from the bottom end, resulting in a fan-like structure of mullite whiskers, as shown in Figure 8C2. A clear disintegration of columnar mullite into needle-like mullite can be observed as the sintering temperature reaches 800 °C (e.g., Figure 9A). More importantly, the morphology of mullite whiskers evolved from secondary growth to anisotropic growth by the time the sintering temperature increased to 825 °C. A dense network structure consisting of uniform mullite crystals was observed when the temperature increased to 825 °C (Figure 9B), and the length and width of mullite whiskers were relatively uniform with a smooth surface, which can be tentatively inferred to be the optimal mullitization reaction at this temperature. The length, width and aspect ratio of mullite whiskers decreased with the increase of the temperature from 800 to 950 °C (Figure 9C–E). It can be inferred that this temperature is higher than the optimal mullitization reaction, which is consistent with the XRD results. It is noteworthy that the anisotropic growth trend of mullite grains is significantly enhanced between 800 °C and 875 °C.

**Figure 10:**
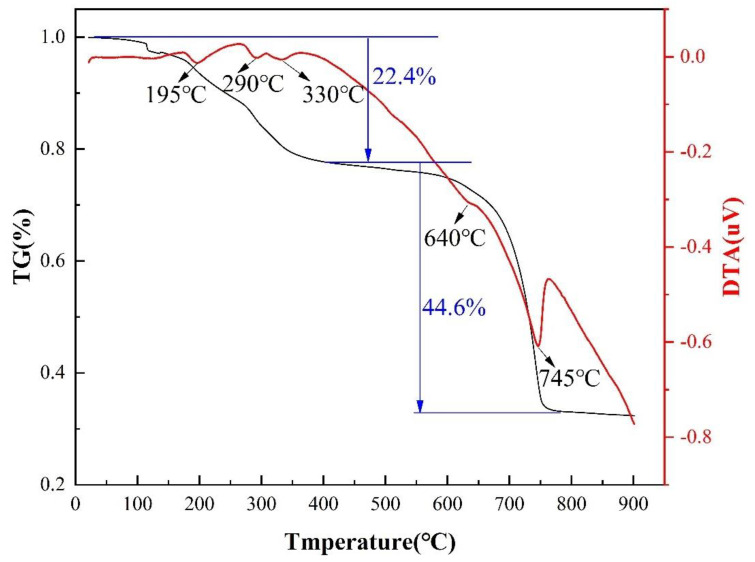
TG-DTA curves of the samples prepared in air from room temperature to 900 °C.

A sample prepared with AlF_3_·3H_2_O assisted with Na_2_SO_4_ molten salt in air is shown in Figure 10 as a TG-DTA curve from room temperature to 900 °C. The TG curve shows a significant weight loss of the sample between 0 and 330 °C with a mass loss of 22.4%. A broad heat absorption peak centered at 195 °C, 290 °C and 330 °C appears on the DTA curve, a phenomenon that can be attributed to the removal of water of crystallization from AlF_3_·3H_2_O and Al_2_(SO_4_)_3_·18H_2_O. The TG curves indicate that the second stage of weight loss started at 640 °C, which is consistent with the relevant literature [44,45,46]. Since the decomposition of Al_2_(SO_4_)_3_ is a heat-absorbing process, the heat-absorbing peak in the corresponding DTA curve appeared at 745 °C. It is concluded from the above analysis that the temperature conditions for stable growth of mullite whiskers are between 720 °C and 900 °C.

**Figure 11:**
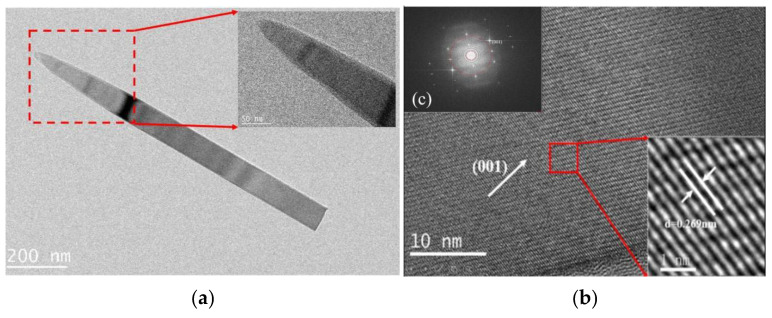
TEM (**a**) HR-TEM (**b**) and SAED (**c**) images of mullite whiskers prepared by sintering at 825 °C for 5 h.

Transmission electron microscopy (TEM), selected area electron diffraction (SAED) and high-resolution transmission electron microscopy (HRTEM) analyses of mullite whiskers prepared by sintering at 825 °C for 5 h are shown in Figure 11. It can be observed that the whiskers have a relatively homogeneous microstructure. Figure 11c shows uniform lattice stripes, indicating an excellent crystallinity of the mullite whiskers, and the lattice stripes show a 0.269 nm crystal plane spacing, which is in good agreement with the lattice stripes of mullite (001) crystal plane (JCPDS No. 15-0776). The results indicate that the synthesized mullite whiskers are perfect single crystals.

### 3.5. Aluminum Fluoride Addition

The growth of mullite whiskers can be effectively promoted by aluminum fluoride trihydrate. The effects of 0.5%, 1%, 2%, 3%, 5% and 7% aluminum fluoride trihydrate additions on the resulting mullite whiskers were analyzed through XRD curves, SEM images and IR spectrum at a fixed sodium sulfate addition of 10% and a sintering temperature of 825 °C.

**Figure 12:**
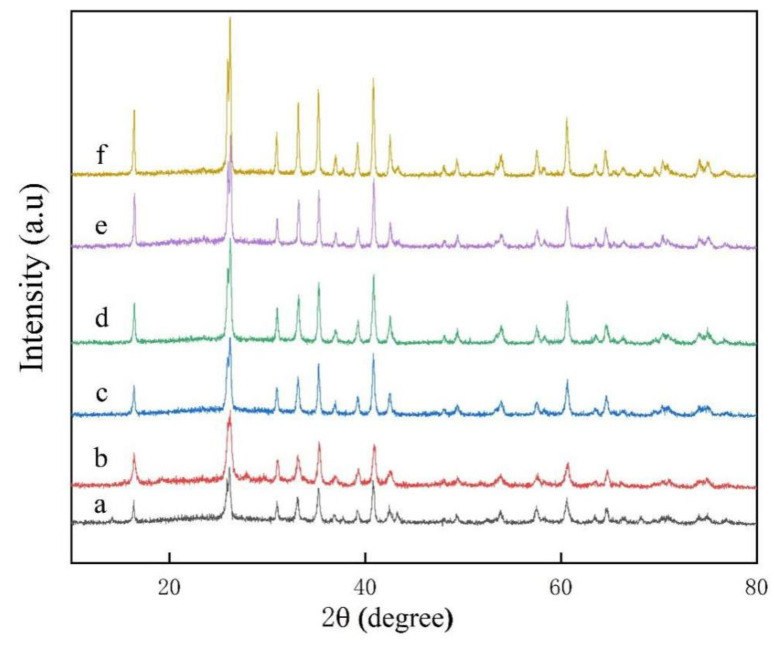
XRD plots of mullite whiskers with different aluminum fluoride contents in 10% sodium sulfate molten salt calcined at 825 °C for 5 h, (a) 0.5% AlF_3_·3H_2_O, (b) 1% AlF_3_·3H_2_O, (c) 2% AlF_3_·3H_2_O, (d) 3% AlF_3_·3H_2_O, (e) 5% AlF_3_·3H_2_O, (f) 7% AlF_3_·3H_2_O.

It can be seen from the XRD curves that the mullite diffraction peaks of the samples undergo a series of phase evolution processes as the content of aluminum fluoride trihydrate increases, as shown in Figure 12. The main crystal diffraction peaks of the XRD curves of the samples at a–f are all mullite (Al_6_Si_2_O_13_, plagioclase, (PDF#15-0076), and no impurity phase generation is ever observed throughout the phase evolution, which indicates that the studied compositions are converted to mullite stoichiometry. On the contrary, the smallest major crystal diffraction peaks of mullite were detected at lower aluminum fluoride trihydrate (a), and the worst crystallinity of mullite crystals in the sample was inferred from the combined width of the diffraction intensities of the crystal phases. However, the intensity of the diffraction peaks of the mullite phase increased rapidly when the aluminum fluoride trihydrate content was further increased to 3–7%. That is the case because the decisive factor that can influence the growth of mullite at the optimum mullite sintering temperature is the content of aluminum fluoride trihydrate. The fluorine atmosphere generated by the high temperature of aluminum trihydrate fluoride promotes faster atomic diffusion, leading to a maximization of the mullite reaction.

**Figure 13:**
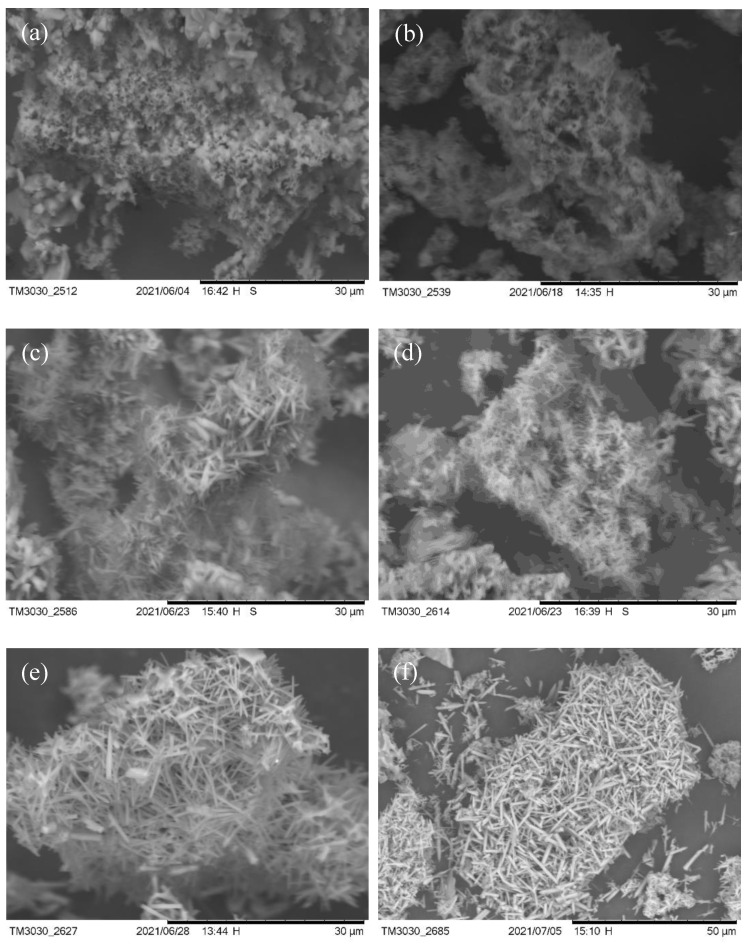
SEM images of mullite whiskers with different aluminum fluoride contents in 10% sodium sulfate molten salt calcined. (**a**) 0.5% AlF_3_·3H_2_O, (**b**) 1% AlF_3_·3H_2_O, (**c**) 2% AlF_3_·3H_2_O, (**d**) 3% AlF_3_·3H_2_O, (**e**) 5% AlF_3_·3H_2_O, (**f**) 7% AlF_3_·3H_2_O.

**Figure 14:**
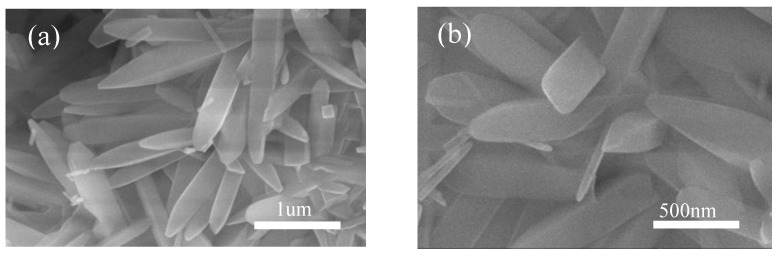
Magnified SEM images of mullite whiskers (5% AlF_3_, 3H_2_O, 825 °C, 5 h). (**a**) for magnification 100,000×; (**b**) for localized mullite whiskers with magnification 200,000×.

It can be clearly seen that many granular mullite crystals are attached to the melt surface, while the particles on the melt surface may be also undeveloped mullite crystalline species, indeterminate SiO_2_ glass phase and amorphous Al_2_O_3_, as shown in Figure 13a. The content of aluminum fluoride trihydrate was added to 1% as shown in Figure 13b, which indicates the further development of mullite crystal species into small-rice-grain size attached to the surface of the composite melt. As Figure 13c shows through the SEM of mullite whiskers prepared by adding 2% aluminum fluoride trihydrate, the whiskers are locally coarsened. By adding 3% aluminum fluoride trihydrate (as shown in Figure 13d), the mullite whiskers developed more uniformly on the basis of (c). The SEM images of the sample with 5% aluminum fluoride trihydrate added are shown in Figure 13e. The needle-like mullite whiskers grew anisotropically and formed a dense meshwork; small catalyst droplets were found on the top of the whiskers, indicating that the liquid phase was also involved in the reaction process. In order to further explore the effect of aluminum fluoride trihydrate on the growth of mullite whiskers, we increased the addition amount to 7% (as shown in Figure 13f). A magnified SEM image of mullite whiskers is shown in Figure 14. The results show that the length of mullite whiskers increased significantly, while the diameter of whiskers increased slowly. It has been reported that aluminum fluoride trihydrate determines the development and growth of mullite, which grows linearly with increasing addition [38,39]. Compared with previous studies on the preparation of mullite whiskers from recycled waste [24,25], the present technique enables the preparation of a large number of mullite whiskers with uniform morphology and a certain interlocking structure and aspect ratio at a lower temperature, which expands the application prospects of whiskers.

**Figure 15:**
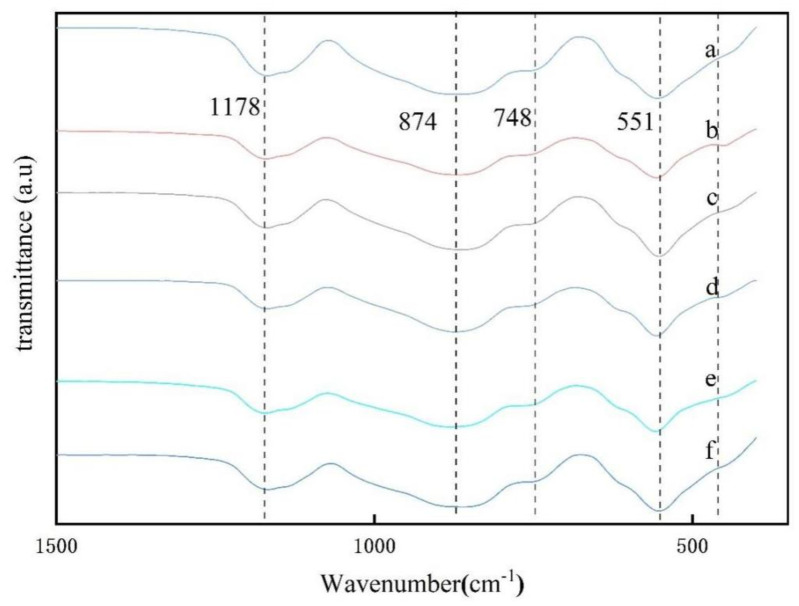
FTIR patterns of mullite whiskers with different aluminum fluoride contents in 10% sodium sulfate molten salt calcined at 825 °C for 5 h for (a) 0.5% AlF_3_·3H_2_O, (b) 1% AlF_3_·3H_2_O, (c) 2% AlF_3_·3H_2_O, (d) 3% AlF_3_·3H_2_O, (e) 5% AlF_3_·3H_2_O, (f) 7% AlF_3_·3H_2_O.

There are almost no bands in the high-frequency region and the mid-frequency region of the FTIR pattern (as shown in Figure 15), but the bands in the low-frequency region are significantly enhanced with the increase of aluminum fluoride trihydrate content, and all samples calcined at 825 °C show broad absorption peaks at 1178 cm^−1^, which can be attributed to the asymmetric vibrational bands of Si-O-Si bonds connected to the [SiO_4_] tetrahedra at the top angle. The FTIR spectra of samples a, b, c, d, e and f were examined and showed that the spectrum bands with mullite characteristics near 551 cm^−1^, 748 cm^−1^, 874 cm^−1^, and 1178 cm^−1^ correspond to the vibrations of AlO_6_, AlO_4_, Al-O-Si, and SiO_4_, respectively [40,47]. These results suggest that the evolution of the transition of amorphous Al_2_O_3_ and SiO_2_ to a crystalline structure already occurs at 825 °C.

### 3.6. Keep-Warm Time

The holding time is also an important factor for whisker growth after determining the proper calcination temperature. The grain will not grow into whiskers with good aspect ratio if the holding time is too short. A longer holding time will not only increase the production cost, but also may make the prepared whiskers coarser, which reduces the quality of the product. To find the optimal time for the growth of mullite whiskers, all other conditions were kept constant and mullite whiskers were prepared by keeping them for 0, 1, 2, 3, 4 and 5 h. The effects of different holding times on whisker growth were analyzed by XRD curves, SEM images and FTIR spectrum.

**Figure 16:**
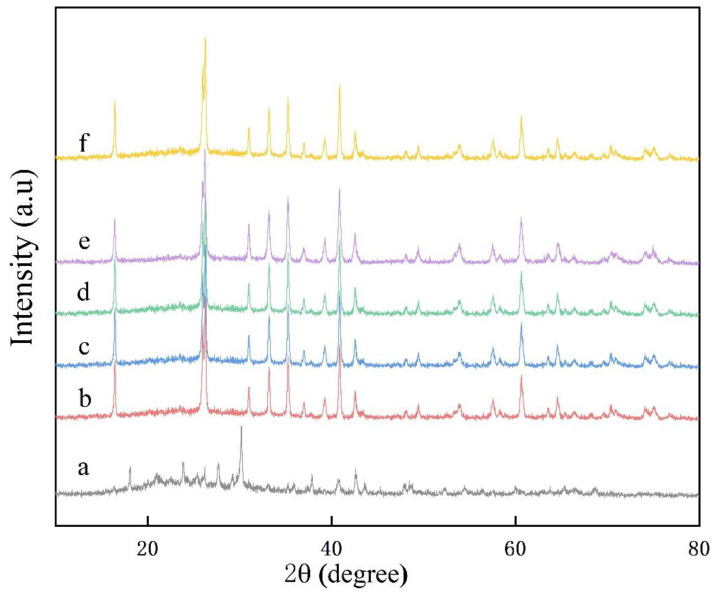
XRD patterns of samples containing 5% aluminum fluoride and 10% sodium sulfate molten salt sintered at 825 °C; (a) holding time is 0 h; (b) 1 h; (c) 2 h; (d) 3 h; (e) 4 h; (f) 5 h.

The XRD patterns of the samples containing 5% aluminum fluoride and 10% sodium sulfate molten salt sintered in 825 °C are represented in Figure 16. It can be seen from the XRD curves that no major crystal diffraction peaks of mullite were detected at lower holding times (a). The major crystal diffraction peaks of the XRD curves of the samples in b–f are all mullite (Al_6_Si_2_O_13_, plagioclase, (PDF#15-0076). The diffraction peak intensity of the mullite phase increased rapidly when the holding time was increased to 1 h. It was inferred from the combined diffraction intensity width of the crystalline phases that the mullite crystals in the samples were well crystallized. That is the case because the decisive factor that can affect the uniform growth of mullite at the optimal mullite sintering temperature and aluminum fluoride trihydrate content is the holding time, in which the fluorine atmosphere generated by the high temperature of aluminum fluoride at a certain time prompts the maximum mullite reaction and the abnormal stabilization of mullitization, leading to a stronger crystallinity of mullite. By comparing the (b)–(f) curves, it was found that the diffraction peak intensities of the (b)–(d) curves do not differ much, which is because the mullite whiskers are still in the development stage and keep forming fine mullite crystalline species in the liquid phase interstices during the holding time of 1–5 h.

**Figure 17:**
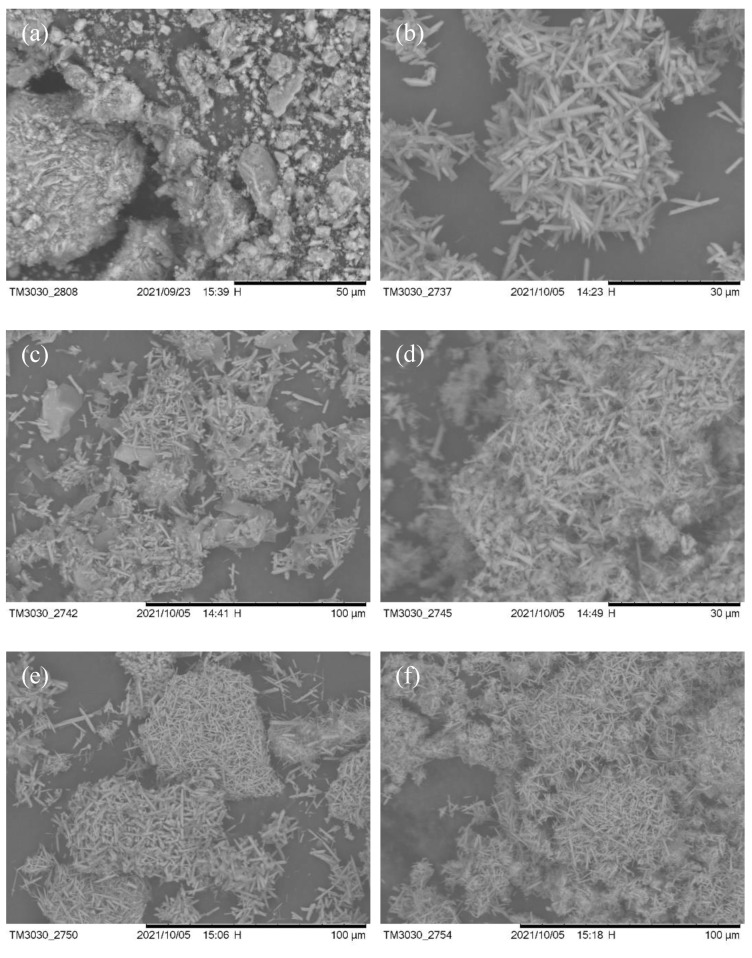
SEM of mullite whisker generation after calcination with different holding times. (**a**) 0 h, (**b**) 1 h, (**c**) 2 h, (**d**) 3 h, (**e**) 4 h, (**f**) 5 h. (10% sodium sulfate, 5% AlF_3_·3H_2_O, 825 °C).

**Figure 18:**
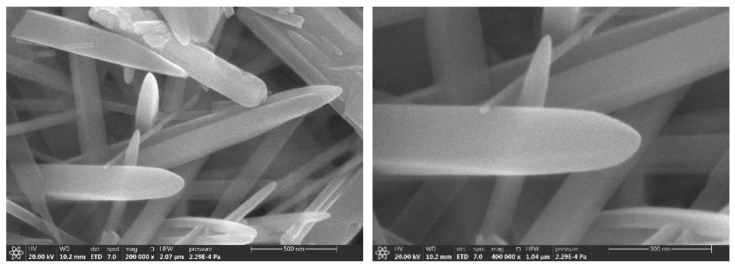
Magnified SEM images of mullite whiskers at 200,000 and 400,000 times (10% sodium sulfate, 5% AlF_3_·3H_2_O, 825 °C, 5 h).

The SEM of the sample held for 0 h is shown in Figure 17a. it can be clearly seen that many granular materials are attached to the entire surface of the melt, where there may be still undeveloped mullite crystal species, indeterminate SiO_2_ glassy phases and amorphous Al_2_O_3_. Mullite crystals rapidly developed into irregular rice-like grains on the basis of the indeterminate small particles in Figure 17a when the holding time was increased to 1 h. At the same time, incomplete reactions of the composite melt and some unwashed molten sodium sulfate salts remained, as shown in Figure 17b. The SEM of the mullite whisker sample prepared by holding for 2 h is shown in Figure 17c. A localized flaky and granular material is clearly visible in the figure, and we infer that this is the stable alumina phase corundum formed during the reaction, and the particles may be indeterminate alumina and mullite. At the same time, mullite becomes coarse and grows unevenly, and there are also molten salts of sodium sulfate attached to the interstitial surface. It indicates that the holding time of 2 h can promote the formation and growth of mullite whiskers with indefinite alumina and indefinite silicon oxide in a fluorine gas environment, and more mullite crystal species are formed around the interstices in a liquid phase environment. Mullite whiskers developed more uniformly when the holding time was 3 h (as shown in Figure 17d). As shown in Figure 17e,f, mullite whiskers are developing more uniformly and mullite whiskers have a denser interlocking structure. The SEM image of the sample kept for 5 h at different magnifications is shown in Figure 18, which forms a good uniform needle-like mullite whisker with anisotropic growth and dense reticulation. Due to the uneven distribution of fluorine atmosphere in the liquid phase environment, the local mullite whiskers were coarsened, but the atmosphere was kept long enough to fully react in the system, and eventually more uniform mullite whiskers were formed. The results showed that the holding time determined the uniformity of mullite development and growth, and the development and growth of mullite increased linearly with the increase of holding time.

**Figure 19:**
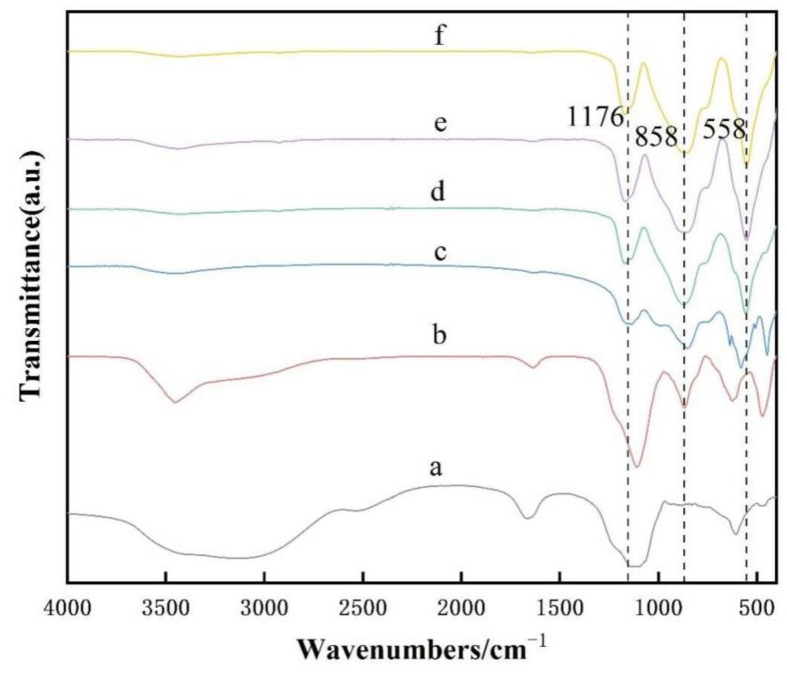
Infrared absorption spectra of mullite whiskers generated after calcination at 825 °C with 10% sodium sulfate molten salt and 5% AlF_3_·3H_2_O catalyst with different holding times, (a) 0 h, (b) 1 h, (c) 2 h, (d) 3 h, (e) 4 h, (f) 5 h.

Figure 19 shows the IR spectrum of the samples heat-treated at different holding times. Curves a and b have a broad strong absorption band in the high-frequency region between 3400 cm^−1^ and 3500 cm^−1^, which is mainly due to OH stretching vibrations. All samples calcined at 825 °C have a broad absorption peak at 1176 cm^−1^, which can be attributed to the asymmetric vibrational band of the Si-O-Si bond of the [SiO_4_] tetrahedron connected to the top corner. The peaks located at 1176 cm^−1^, 858 cm^−1^ and 558 cm^−1^ show the characteristic bands of mullite, which correspond to SiO_4_, AlO_4_ and AlO_6_ stretching vibrations.

## 4. Conclusions

In this paper, mullite whiskers were successfully prepared by the fluorine gas-assisted molten salt method using industrial silica-rich waste as raw material. The influencing factors of the synthesized whiskers were analyzed by various characterization methods, and the effects of rare earth elements, Na_2_SO_4_ content, aluminum fluoride addition in the raw material, different sintering temperatures and holding times on the properties of the resulting whiskers were analyzed. According to the results obtained from the conducted studies, we can mention the following conclusions:

1. The rare earth elements in the raw material and the use of 10% Na_2_SO_4_ as flux promote the growth of nuclei and accelerate the crystallization rate.
2. The addition of 5% aluminum fluoride resulted in stronger crystallinity and more homogeneous morphology of mullite.
3. The optimal growth temperature of mullite whiskers is 825 °C.
4. The optimal holding time after calcination is 5 h.

The whisker prepared in this paper is high-quality with high-temperature resistance and perfect crystallization, and can be widely used as a reinforcement material for ceramic-based, metal-based and plastic-based composites. In addition, the mullite whiskers prepared in this study exhibit a white powder after washing and drying. In contrast, commercially available mullite powder is usually obtained by crushing the solid sinter, which is expensive and requires further processing costs. The reason for the very low target cost of mullite whiskers prepared using this technique is that the silica-rich waste itself has no economic value. Moreover, the low additive content used in this study and the large number of mullite whiskers produced at a low temperature can significantly reduce the cost. The raw materials required for this experiment were obtained from industrial waste, and the mullite whiskers were prepared by a simple process, which is expected to be transferable to the industrial production of mullite whiskers.

## Figures and Tables

**Table 1:** Chemical composition (wt%) analysis table of each component of silica-rich waste powder.

Al_2_O_3_ wt%	SiO_2_ wt%	Na_2_O wt%	SO_3_ wt%	La_2_O_3_ wt%	CeO_2_ wt%	MgO wt%
26.2	48.7	5.8	3.9	5.6	6.4	3.4

**Table 2:** Chemical composition (wt%) analysis table for each component of pretreated silica-rich waste powder with diluted rare earth oxides removed.

Al_2_O_3_ wt%	SiO_2_ wt%	Na_2_O wt%	SO_3_ wt%	La_2_O_3_ wt%	CeO_2_ wt%	MgO wt%
28.9	56.7	6.3	4.4	0.05	0.05	3.6

## Data Availability

Data available on request due to restrictions e.g., privacy or ethical.

## References

[1] Schneider H., Fischer R.X., Schreuer J. (2015). Mullite: Crystal Structure and Related Properties. J. Am. Ceram. Soc..

[2] Zhu L., Li S., Gao Z., Zhang X., Zhang L., Li H., Liu G. (2023). Effect of in situ formed acicular mullite whiskers on thermal shock resistance of alumina-mullite refractories. J. Aust. Ceram. Soc..

[3] Deng X., Ji P., Yin J., Wang Y., Song C., Li S., Zhang Y., Ding X., Ran S., Zhang H. (2022). Fabrication and characterization of mullite-whisker-reinforced lightweight porous materials with AlF_3_·3H_2_O. Ceram. Int..

[4] Zhao P., Ma S., Wang X., Wu W., Ou Y. (2023). Properties and mechanism of mullite whisker toughened ceramics. Ceram. Int..

[5] Liu Z., Yu J., Wang X., Wang J., Zhang L., Wen T., Jia D., Yan Z., Yuan L., Ma B. (2022). Using Si/SiC solid waste to design urchin-like mullite whiskers for oil-water separation. Int. J. Appl. Ceram. Technol..

[6] Dong X., Zheng Y., Xie D. (2023). Multi-functional mullite fiber-based porous ceramics with a multilevel pore structure assembled by alumina platelets and mullite whiskers. Ceram. Int..

[7] Yang P., Liu S., Mao Z., Wang D. (2023). Preparation and properties of hierarchical structural alumina/mullite composites. J. Eur. Ceram. Soc..

[8] Schneider H., Schreuer J., Hildmann B. (2008). Structure and properties of mullite—A review. J. Eur. Ceram. Soc..

[9] Yang F., Zhao S. (2023). Fibrous porous mullite ceramics modified by mullite whiskers for thermal insulation and sound absorption. J. Eur. Ceram. Soc..

[10] Yang Z., Yang F., Zhao S. (2021). In-situ growth of mullite whiskers and their effect on the microstructure and properties of porous mullite ceramics with an open/closed pore structure. J. Eur. Ceram. Soc..

[11] Mahnicka-Goremikina L., Svinka R., Svinka V. (2022). Thermal Properties of Porous Mullite Ceramics Modified with Microsized ZrO_2_ and WO_3_. J. Mater..

[12] Shin C., Oh S.-H., Choi J.-H. (2021). Synthesis of porous ceramic with well-developed mullite whiskers in system of Al_2_O_3_-Kaolin-MoO_3_. J. Mater. Res. Technol..

[13] Li K., Ge S., Yuan G., Zhang H., Zhang J., He J., Jia Q., Zhang S. (2020). Effects of V_2_O_5_ addition on the synthesis of columnar self-reinforced mullite porous ceramics. Ceram. Int..

[14] Zhang C., Lu S., Zhang Z., Zhong M., Li Y., Huang X., Xu W., Wang D., Wang L. (2018). The effect of phosphorus on the formation of mullite whiskers from citric acid activated kaolin. Ceram. Int..

[15] Song X., Liu W., Xu S. (2018). Microstructure and elastic modulus of electrospun Al_2_O_3_-SiO_2_-B_2_O_3_ composite nanofibers with mullite-type structure prepared at elevated temperatures. J. Eur. Ceram. Soc..

[16] Kim B., Cho Y., Yoon S., Stevens R., Park H. (2009). Mullite whiskers derived from kaolin. Ceram. Int..

[17] Alves H.P., Silva J.B., Campos L.F., Torres S.M., Dutra R.P., Macedo D.A. (2016). Preparation of mullite based ceramics from clay–kaolin waste mixtures. Ceram. Int..

[18] Bella M., Hamidouche M., Gremillard L. (2021). Preparation of mullite-alumina composite by reaction sintering between Algerian kaolin and amorphous aluminum hydroxide. Ceram. Int..

[19] Hua K., Shui A., Xu L., Zhao K., Zhou Q., Xi X. (2016). Fabrication and characterization of anorthite–mullite–corundum porous ceramics from construction waste. Ceram. Int..

[20] Xu X., Liu X., Wu J., Zhang C., Zhang Q., Tian K. (2020). Preparation of mullite whisker reinforced SiC membrane supports with high gas permeability. Ceram. Int..

[21] Li L., Cao G., Zhao R., Wang L., Wu S. (2019). High-porosity whisker-mullite/corundum membrane support prepared from recycled industrial waste coal cinder. Ceram. Int..

[22] Zawrah M.F., Wassel A.R. (2022). Recycling of aluminum dross and silica fume wastes for production of mullite-containing ceramics: Powder preparation, sinterability and properties. Ceram. Int..

[23] Liu J., Xu J., Zhang Y., Su Z., Xu C., Jiang T. (2022). Co-utilization of secondary aluminum dross and ferronickel slag for preparation of cordierite–mullite insulating ceramic. J. Am. Ceram. Soc..

[24] Fu M., Liu J., Dong X., Zhu L., Dong Y., Hampshire S. (2019). Waste recycling of coal fly ash for design of highly porous whisker-structured mullite ceramic membranes. J. Eur. Ceram. Soc..

[25] Das D., Nijhuma K., Gabriel A.M., Daniel G.P.F., Murilo D.D.M.I. (2020). Recycling of coal fly ash for fabrication of elongated mullite rod bonded porous SiC ceramic membrane and its application in filtration. J. Eur. Ceram. Soc..

[26] Yang H.-L., Li Z.-S., Ding Y.-D., Ge Q.-Q., Shi Y.-J., Jiang L. (2022). Effect of Silicon Source (Fly Ash, Silica Dust, Gangue) on the Preparation of Porous Mullite Ceramics from Aluminum Dross. Materials.

[27] Liu M., Zhu Z., Zhang Z., Chu Y., Yuan B., Wei Z. (2019). Development of highly porous mullite whisker ceramic membranes for oil-in-water separation and resource utilization of coal gangue. Sep. Purif. Technol..

[28] Liu R., Xiang D. (2021). Recycling photovoltaic silicon waste for fabricating porous mullite ceramics by low-temperature reaction sintering. J. Eur. Ceram. Soc..

[29] Xing Z., Hu Y., Xiang D. (2020). Porous SiC-mullite ceramics with high flexural strength and gas permeability prepared from photovoltaic silicon waste. Ceram. Int..

[30] Li S., Du H., Guo A., Xu H., Yang D. (2012). Preparation of self-reinforcement of porous mullite ceramics through in situ synthesis of mullite whisker in fly ash body. Ceram. Int..

[31] Garai M., Karmakar B. (2016). Rare earth ion controlled crystallization of mica glass-ceramics. J. Alloy Compd..

[32] Liu H., Zou X., Wang X., Lu X., Ding W. (2012). Effect of CeO_2_ addition on Ni/Al_2_O_3_ catalysts for methanation of carbon dioxide with hydrogen. J. Nat. Gas Chem..

[33] Luo S., Shi Z., Li N., Lin Y., Liang Y., Zeng Y. (2019). Crystallization inhibition and microstructure refinement of Al-5Fe alloys by addition of rare earth elements. J. Alloy Compd..

[34] Feng Z., Wang M., Lu R., Xu W., Zhang T., Wei T., Zhang J., Liao Y. (2020). A composite structural high-temperature-resistant adhesive based on in-situ grown mullite whiskers. Mater. Today Commun..

[35] Huang X., Zhao D., Ma L., Deng C., Li L., Chen K., Yang X. (2020). Effect of La_2_O_3_ on crystallization of Glass-ceramics. J. Non-Cryst. Solids.

[36] Wang M., Fang L., Li M. (2019). Phase separation and crystallization of La_2_O_3_ doped ZnO-B_2_O_3_-SiO_2_ glass. J. Rare Earths.

[37] Qi J., Liu C., Liu H., Li C., Jiang M. (2021). Effect of rare earth oxide on the crystallization behavior of CaO-Al_2_O_3_-based mold flux for rare earth heat-resistant steel continuous casting. J. Non-Cryst. Solids.

[38] Abdullayev A., Klimm D., Kamutzki F., Gurlo A., Bekheet M.F. (2021). AlF_3_-assisted flux growth of mullite whiskers and their application in fabrication of porous mullite-alumina monoliths. Open Ceram..

[39] Chen P., Gu X., Liu S., Zhu S., Wang T., Zhu Y., Fan A., Liu Y. (2023). Industrial waste silica-alumina gel recycling: Low-temperature synthesis of mullite whiskers for mass production. Ceram. Int..

[40] Baccour A., Sahnoun R.D., Bouaziz J. (2014). Effects of mechanochemical treatment on the properties of kaolin and phosphate–kaolin materials. Powder Technol..

[41] Sembiring S., Simanjuntak W., Manurung P. (2014). Synthesis and characterisation of gel-derived mullite precursors from rice husk silica. J. Ceram. Int..

[42] Feng G., Jiang F., Jiang W. (2018). Novel facile nonaqueous precipitation in-situ synthesis of mullite whisker skeleton porous materials. Ceram. Int..

[43] Zhao H., Li X., Ji H., Yu H., Yu B., Qi T. (2018). Constructing secondary-pore structure by in-situ synthesized mullite whiskers to prepare whiskers aerogels with ultralow thermal conductivity. J. Eur. Ceram. Soc..

[44] Yang T., Peng-long Q., Mei Z. (2015). Molten salt synthesis of mullite nanowhiskers using different silica sources. J. Int. Miner. Metall. Mater..

[45] Liu S., Liu J., Du H., Hou F., Guo A. (2014). Microstructure of mullite fiber-based hierarchical structures adjusted by Al/Si mole ratio of the raw material powders. Ceram. Int..

[46] Zheng G., Xia J., Chen Z. (2020). Thermodynamics and kinetics of the carbothermal reduction of aluminum sulfate. Phosphorus Sulfur Silicon Relat. Elem..

[47] Wu L., Li C., Chen Y. (2021). Seed assisted in-situ synthesis of porous anorthite/mullite whisker ceramics by foam-freeze casting. J. Ceram. Int..

